# Virtual pacing of a patient’s digital twin to predict left ventricular reverse remodelling after cardiac resynchronization therapy

**DOI:** 10.1093/europace/euae009

**Published:** 2024-01-30

**Authors:** Tijmen Koopsen, Willem Gerrits, Nick van Osta, Tim van Loon, Philippe Wouters, Frits W Prinzen, Kevin Vernooy, Tammo Delhaas, Arco J Teske, Mathias Meine, Maarten J Cramer, Joost Lumens

**Affiliations:** Department of Biomedical Engineering, CARIM Cardiovascular Research Institute Maastricht, Maastricht University, Universiteitssingel 40, 6200 MD, The Netherlands; Department of Cardiology, Division of Heart and Lungs, University Medical Center Utrecht (UMCU), Utrecht, The Netherlands; Department of Biomedical Engineering, CARIM Cardiovascular Research Institute Maastricht, Maastricht University, Universiteitssingel 40, 6200 MD, The Netherlands; Department of Biomedical Engineering, CARIM Cardiovascular Research Institute Maastricht, Maastricht University, Universiteitssingel 40, 6200 MD, The Netherlands; Department of Cardiology, Division of Heart and Lungs, University Medical Center Utrecht (UMCU), Utrecht, The Netherlands; Department of Physiology, CARIM Cardiovascular Research Institute Maastricht, Maastricht University, Maastricht, The Netherlands; Department of Cardiology, CARIM Cardiovascular Research Institute Maastricht, Maastricht University, Maastricht, The Netherlands; Department of Cardiology, Maastricht University Medical Center (MUMC), Maastricht, The Netherlands; Department of Cardiology, Radboud University Medical Center, Nijmegen, The Netherlands; Department of Biomedical Engineering, CARIM Cardiovascular Research Institute Maastricht, Maastricht University, Universiteitssingel 40, 6200 MD, The Netherlands; Department of Cardiology, Division of Heart and Lungs, University Medical Center Utrecht (UMCU), Utrecht, The Netherlands; Department of Cardiology, Division of Heart and Lungs, University Medical Center Utrecht (UMCU), Utrecht, The Netherlands; Department of Cardiology, Division of Heart and Lungs, University Medical Center Utrecht (UMCU), Utrecht, The Netherlands; Department of Biomedical Engineering, CARIM Cardiovascular Research Institute Maastricht, Maastricht University, Universiteitssingel 40, 6200 MD, The Netherlands

**Keywords:** *In silico* clinical trial, Left bundle branch block, CircAdapt, Myocardial work, Patient-specific modelling, Computer modelling and simulation

## Abstract

**Aims:**

Identifying heart failure (HF) patients who will benefit from cardiac resynchronization therapy (CRT) remains challenging. We evaluated whether virtual pacing in a digital twin (DT) of the patient’s heart could be used to predict the degree of left ventricular (LV) reverse remodelling post-CRT.

**Methods and results:**

Forty-five HF patients with wide QRS complex (≥130 ms) and reduced LV ejection fraction (≤35%) receiving CRT were retrospectively enrolled. Echocardiography was performed before (baseline) and 6 months after CRT implantation to obtain LV volumes and 18-segment longitudinal strain. A previously developed algorithm was used to generate 45 DTs by personalizing the CircAdapt model to each patient’s baseline measurements. From each DT, baseline septal-to-lateral myocardial work difference (MW_LW-S,DT_) and maximum rate of LV systolic pressure rise (dP/dt_max,DT_) were derived. Biventricular pacing was then simulated using patient-specific atrioventricular delay and lead location. Virtual pacing–induced changes ΔMW_LW-S,DT_ and ΔdP/dt_max,DT_ were correlated with real-world LV end-systolic volume change at 6-month follow-up (ΔLVESV). The DT’s baseline MW_LW-S,DT_ and virtual pacing–induced ΔMW_LW-S,DT_ were both significantly associated with the real patient’s reverse remodelling ΔLVESV (*r* = −0.60, *P* < 0.001 and *r* = 0.62, *P* < 0.001, respectively), while correlation between ΔdP/dt_max,DT_ and ΔLVESV was considerably weaker (*r* = −0.34, *P* = 0.02).

**Conclusion:**

Our results suggest that the reduction of septal-to-lateral work imbalance by virtual pacing in the DT can predict real-world post-CRT LV reverse remodelling. This DT approach could prove to be an additional tool in selecting HF patients for CRT and has the potential to provide valuable insights in optimization of CRT delivery.

## Graphical Abstract

**Graphical Abstract:**
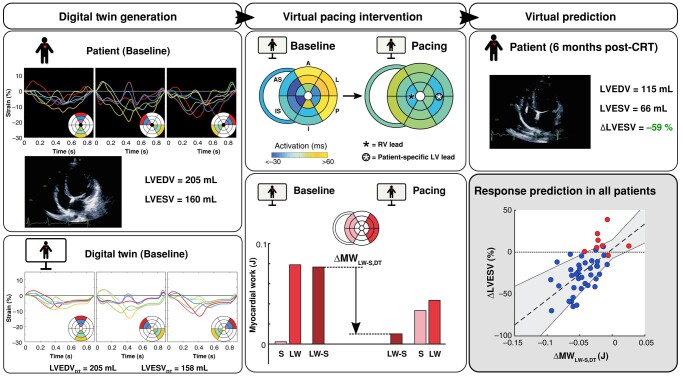

**What’s new?**

- Imaging-based digital twins were created of 45 heart failure patients treated with cardiac resynchronization therapy.
- Virtual pacing intervention in the digital twins revealed a direct correlation between its mechanical response and the actual degree of left ventricular reverse remodelling observed in patients during follow-up.
- Future investigations should explore the capacity of this digital twin methodology to enhance patient outcomes by predicting the optimal strategy for pacing delivery tailored to each individual.
- The study underscores the potential of the CircAdapt model of the human heart and circulation as a virtual platform for *in silico* (pre-)clinical trials in virtual heart failure patients.

## Introduction

The goal of cardiac resynchronization therapy (CRT) is to improve left ventricular ejection fraction (LVEF), reduce symptoms, and improve survival of patients with dyssynchronous heart failure (HF).^1,2^ However, despite the importance of CRT as a HF treatment, a substantial number of implanted patients show no improvements.^3^ At the same time, CRT is widely underutilized in eligible patients.^4^ Uncertainty of which patients will benefit remains a challenge that affects the full potential of this HF therapy.^4,5^ To improve patient selection, by taking into account more than the standard LVEF and electrocardiographic characteristics,^6^ a personalized modelling approach using the patient’s digital twin (DT) may provide important insights.^7^

The DT is a virtual representation of the patient’s heart, obtained from integrating a set of patient-specific measurements into a computer model.^8^ Thereby, the DT describes the unique pathophysiology of the patient and can function as a platform to evaluate the effect of therapeutic interventions. Based on the diagnostic value of myocardial strain measurements in dyssynchronous HF patients,^9–11^ we have previously developed an algorithm that uses left ventricular (LV) strain and volume measurements to generate a DT.^12^

In this study, we evaluate whether virtual pacing of these DTs can be used to predict the degree of post-CRT LV reverse remodelling. Hereto, we retrospectively perform simulations of biventricular pacing as clinically administered in a population of dyssynchronous HF patients, including personalized atrioventricular (AV) delay and lead location. Based on previous findings suggesting that acute recoordination is more strongly associated with long-term LV reverse remodelling than baseline dyssynchrony only,^13,14^ we quantify the pacing-induced reduction of septal-to-lateral work difference in the DT as a potential predictor of LV reverse remodelling, as well as acute haemodynamic improvement.

## Methods

### Study population

We retrospectively included 45 HF patients with a QRS width ≥120 ms and a reduced LVEF (≤35%), all previously selected for CRT device implantation. Patients were randomly selected from three different studies^13,15,16^ based on availability and quality of all three echocardiographic views to allow derivation of 18-segment longitudinal strain. All three studies were approved by the institutional review boards or local medical ethics committees of the participating centres, and informed consent was obtained from all study participants. Characteristics of the patient population are shown in *Table 1*.

**Table 1:** Clinical characteristics of the patient population (*n* = 45)

Age (years)	66 ± 10
Male gender (%, *n*)	62%, 28
QRS duration (ms)	171 ± 21
LBBB morphology^a^ (%, *n*)	84%, 38
CRT Class I indication (%, *n*)	82%, 37
Atrial fibrillation (%, *n*)	11%, 5
Ischaemic heart disease (%, *n*)	33%, 15
LVEDV (mL)	217 ± 83
LVESV (mL)	172 ± 81
LVEF (%)	23 ± 9
ACE-inhibitor/AT2 (%, *n*)	93%, 42
Beta-blocker (%, *n*)	67%, 30
Diuretics (%, *n*)	96%, 43
Spironolactone/eplerenone (%, *n*)	51%, 23

ACE, angiotensin converting enzyme; AT2, angiotensin-2; CRT, cardiac resynchronization therapy; LBBB, left bundle branch block; LVEDV, left ventricular end-diastolic volume; LVEF, left ventricular ejection fraction; LVESV, left ventricular end-systolic volume.

^a^Based on the Strauss criteria for LBBB.^17^

### Echocardiography

Routine echocardiographic imaging was performed at baseline and 6 months after CRT device implantation (mean follow-up: 6.4 ± 1.4 months) using a Vivid E9 or E95 Ultrasound system (GE Healthcare, Horten, Norway). All images were digitally stored and analysed offline. Left ventricular end-diastolic and end-systolic volumes were calculated using biplane Simpson’s method and were used to calculate the relative change of LV end-systolic volume from baseline to follow-up (ΔLVESV). Good quality baseline two-chamber, three-chamber, and four-chamber echocardiographic acquisitions were used for speckle tracking analysis (EchoPac version 203) to obtain LV 18-segment longitudinal strain. Regions of interest were automatically tracked and manually adjusted to the endo- and epicardial border following the standard recommendations.^18^ Timing of mitral valve closure was derived from Doppler flow tracings and selected as the moment of zero-strain reference.

### Cardiac resynchronization therapy

Cardiac resynchronization therapy devices were implanted according to local implantation protocols under local anaesthesia.^13,15,16^ Right ventricular (RV) and right atrial leads were placed transvenously at conventional positions. Taking into account adequate pacing thresholds and absence of phrenic nerve stimulation, the LV lead was preferably placed in a lateral or posterolateral vein. Right ventricular leads were positioned preferably in the RV apex. Although no uniform AV and ventriculo-ventricular (VV) delays were mandated, paced AV delay was set to 90–130 ms, and VV delay was set to either 0 or −40 ms (LV first).

### Digital twin generation algorithm

**Figure 1:**
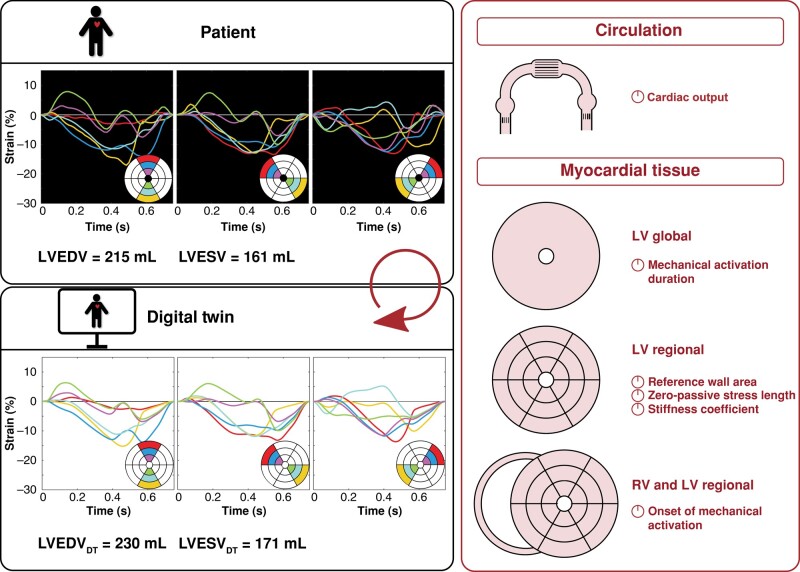
Overview of the input and output of the digital twin generation algorithm.^12^ Left ventricular 18-segment longitudinal strain, LVEDV, and LVESV measurements of the patient (upper left panel) were used to automatically estimate cardiac output, global LV mechanical activation duration, regional LV myocardial constitutive properties, and regional LV and RV onset of mechanical activation (right panel). As a result, a personalized model (DT) was obtained, which resembled the patient (lower left panel). DT, digital twin; LV, left ventricular; LVEDV, left ventricular end-diastolic volume; LVESV, left ventricular end-systolic volume; RV, right ventricular.

For each patient, a DT was automatically generated using a previously developed algorithm that personalized the CircAdapt cardiovascular model.^12^ Details on the DT generation algorithm and on the model equations can be found in the Supplementary material. In short, this algorithm used the patient’s baseline measurements of LV 18-segment longitudinal strain, end-diastolic volume (EDV), and end-systolic volume (ESV) and was blinded to follow-up data. The algorithm utilized dynamic multi-swarm particle swarm optimization (DMS-PSO) to minimize the mean squared error between measured and simulated LV strain and volumes. To calculate this mean squared error, the difference between measured and simulated strain and strain rate as well as the difference between measured and simulated LVEDV and LVEF were normalized to their expected measurement uncertainties. In total, 75 model parameters describing cardiac output, global LV activation duration, AV delay, and LV regional myocardial constitutive properties were selected based on an objective parameter subset reduction pipeline^12^ and optimized to personalize parameter values (*Figure 1*). As a result, the patient’s unique DT was obtained.

### Digital twin: baseline indices

Maximum rate of LV systolic pressure rise (dP/dt_max,DT_) and septal-to-lateral myocardial work difference (MW_LW-S,DT_) of the baseline DT were calculated. Segmental MW was calculated as the area of the regional stress–strain loop multiplied with segmental wall volume. Value of MW_LW-S,DT_ was calculated as the absolute difference between average MW of the four basal and mid-ventricular lateral and posterior wall segments and average MW of the four basal and mid-ventricular anteroseptal and inferoseptal segments.

### Virtual pacing and derived indices

**Figure 2:**
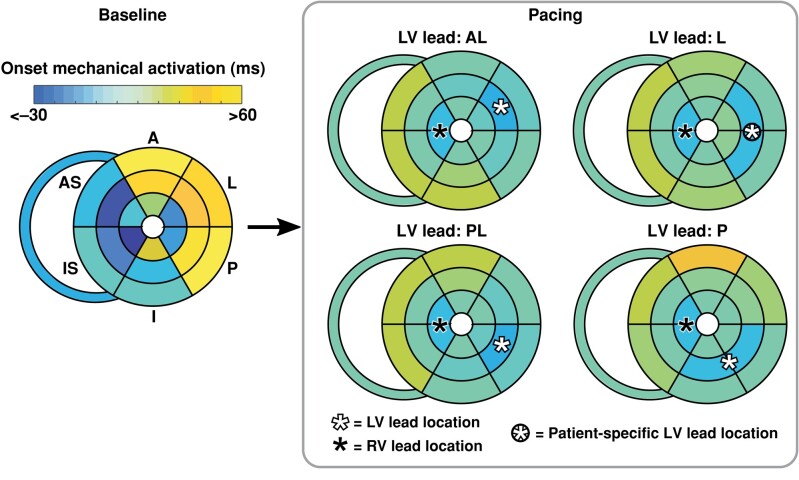
Bullseye plots of RV and LV regional mechanical activation at baseline and during virtual biventricular pacing in the DT. Starting from the baseline DT with automatically estimated onset of activation in the RV wall and LV wall segments (Baseline), a personalized pacing configuration was simulated in which activation was initiated at the location of the RV and patient-specific LV lead, indicated by the black and white asterisks, respectively (Pacing). From the pacing lead locations, activation propagated radially with an intersegmental delay of 15 ms. The patient illustrated here had a L LV lead location, indicated by the circled asterisk. Furthermore, patient-specific AV delay was set to 130 ms based on the pacing protocol of the original study. Note that definition of myocardial segment location differs slightly from that of LV lead location. A, anterior; AL, anterolateral; AS, anteroseptal; I, inferior; IS, inferoseptal; L, lateral; LV, left ventricular; P, posterior; PL, posterolateral; RV, right ventricular.

Starting from the baseline DT, a patient-specific biventricular pacing intervention was simulated in which activation propagated radially from the location of the RV and LV leads with an intersegmental delay of 15 ms (*Figure 2*). While the location of the RV lead was similar in all patients and therefore fixed, LV lead location differed between patients. Therefore, four different patient-specific LV lead locations were simulated, i.e. anterolateral (AL), lateral (L), posterolateral (PL), and posterior (P). Furthermore, AV delay was set to either 130^16^ or 100 ms^13,15^ depending on the pacing protocol of the original study. During pacing, activation of the RV wall was assumed to equal the average activation time of the septum. Absolute changes of MW_LW-S,DT_ (ΔMW_LW-S,DT_) and dP/dt_max,DT_ (ΔdP/dt_max,DT_) were calculated.

### Statistical analysis

Analysis was performed using MATLAB R2022b (MathWorks, Natick, MA, USA). The relations between baseline MW_LW-S,DT_, ΔMW_LW-S,DT_, ΔdP/dt_max,DT_, and ΔLVESV were assessed by univariate linear regression analysis with calculation of Pearson’s correlation coefficient (*r*) and associated *P*-values.

## Results

### Digital twin generation

The patient’s echocardiographic volume and strain data were well reproduced by their DTs. In summary, LVEDV and LVESV differed from the measurements by 8 ± 7 and 5 ± 5 mL, respectively, while average deviation of strain per measurement point during the cardiac cycle was 1.5 ± 0.7%.

### Virtual pacing and work redistribution

**Figure 3:**
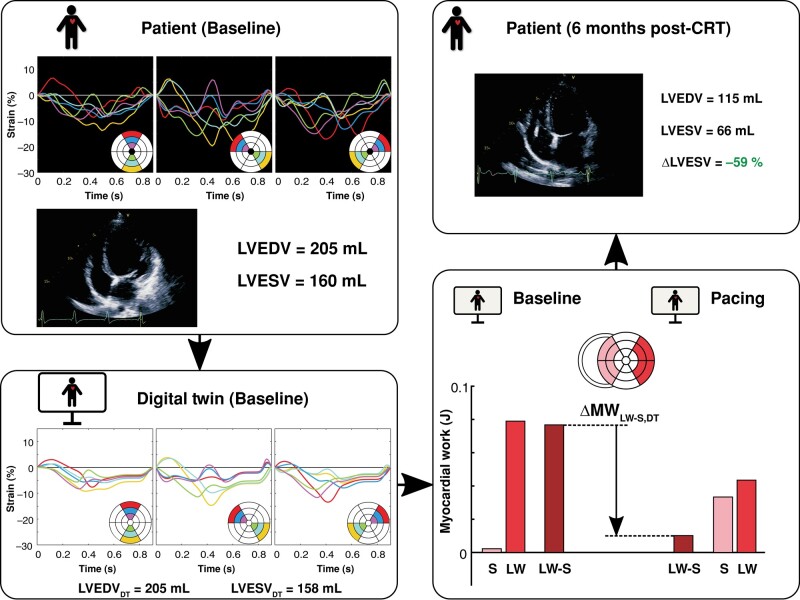
The association between septal-to-lateral work imbalance reduction by virtual pacing in the DT and long-term clinical LV reverse remodelling after CRT device implantation, illustrated for a single patient with lateral LV lead location. The DT of this patient demonstrated a relatively large pacing-induced reduction of septal-to-lateral myocardial work difference (ΔMW_LW-S,DT_), which corresponded with a relatively large reduction of LV end-systolic volume (ΔLVESV) in the patient at clinical follow-up. CRT, cardiac resynchronization therapy; LVEDV, left ventricular end-diastolic volume; LVESV, left ventricular end-systolic volume; LW, lateral wall; S, septum.

For a single patient, *Figure 3* shows the LV volumes and regional strain of the baseline DT, as well as its MW_LW-S,DT_ at baseline and after simulating pacing. It can be observed that the septum produced almost zero work at baseline, while the LV lateral wall generated a large amount of positive work. Consequently, MW_LW-S,DT_ had a large positive value at baseline. During pacing, septal work was increased while LV lateral wall work was reduced, leading to a relatively large reduction of MW_LW-S,DT_. During clinical follow-up, the patient demonstrated a ΔLVESV of −59%.

**Figure 4:**
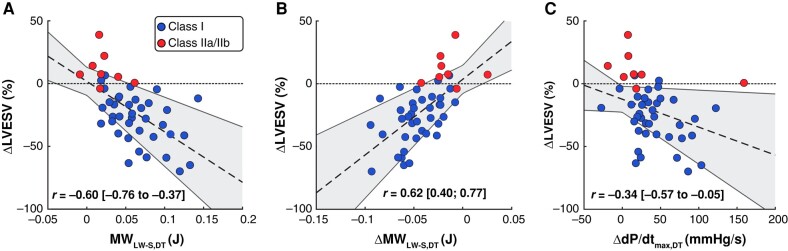
Correlation plots of relative LV volume change 6 months after CRT in the patient (ΔLVESV) vs. baseline DT septal-to-lateral myocardial work difference (MW_LW-S,DT_, *A*) and indices derived from virtual pacing of the DT: absolute change in MW_LW-S,DT_ (ΔMW_LW-S,DT_, *B*), and absolute change of maximum rate of LV systolic pressure rise (ΔdP/dt_max,DT_, *C*). The blue dots (*n* = 37) represent patients with a Class I indication for CRT, while the red dots (*n* = 8) represent patients with a Class IIa or IIb indication for CRT, according to the Strauss criteria for LBBB.^17^ Indices MW_LW-S,DT_ and ΔMW_LW-S,DT_ were significantly associated with ΔLVESV (*r* = −0.60, *P* < 0.001 and *r* = 0.62, *P* < 0.001, respectively), while ΔdP/dt_max,DT_ correlated considerably weaker with ΔLVESV (*r* = −0.34, *P* = 0.02).

For the whole patient population, baseline MW_LW-S,DT_ and ΔMW_LW-S,DT_ correlated moderately with ΔLVESV (*r* = −0.60, *P* < 0.001 and *r* = 0.62, *P* < 0.001, respectively; *Figure 4*). At the same time, ΔdP/dt_max,DT_ correlated weaker with ΔLVESV (*r* = −0.34, *P* = 0.02).

## Discussion

The present study investigated whether virtual pacing in DTs of HF patients, generated using baseline echocardiographic imaging data, could be used to predict the degree of LV reverse remodelling 6 months after CRT. It was found that the virtual pacing–induced reduction of septal-to-lateral work difference in the DT was significantly associated with the patient’s LVESV reduction at clinical follow-up. This DT technology could prove to be an additional tool in selecting HF patients for CRT and has the potential to provide valuable insights in optimization of CRT delivery.

Virtual pacing of our DTs revealed that the DT’s acute mechanical recoordination was more strongly associated with the patient’s LVESV reduction than the DT’s acute haemodynamic improvement. This result agrees with the findings of recent clinical studies^13,14^ and suggests that mechanical recoordination can be an effective target for optimizing pacing delivery, also in the DT. Acute haemodynamic improvement represents the acute success of resynchronization but its predictive value for long-term LV reverse remodelling varies among studies.^19,20^

The DT’s baseline septal-to-lateral work imbalance as well as its change after virtual pacing correlated well with real-world LV reverse remodelling (*Figure 4*). Firstly, this corroborates previous clinical studies showing that baseline septal-to-lateral work imbalance is a marker of CRT response potential.^14,21^ Secondly, this finding suggests that our virtual pacing interventions were representative for clinical CRT, thereby strengthening the credibility of our DT technology.

Interestingly, the DTs of the few patients with Class II indications according to the 2013 ESC Guidelines on cardiac pacing and CRT^22^ showed a small reduction of septal-to-lateral work difference by virtual pacing as compared with the DTs of the patients with a Class I indication. Even more interestingly, the virtual pacing effect appeared to have additional prognostic value in patients with a Class I indication for CRT. Within this subgroup, there was significant heterogeneity in the degree of LV reverse remodelling, which was strongly associated with the virtual pacing–induced work redistribution in their DTs.

The comparison of DT-derived indices with measures of long-term LV reverse remodelling is supported by the good correlation of LVESV reduction with mortality.^23^ Furthermore, a similar association between acute MW redistribution by CRT and LV reverse remodelling has previously been established in clinical studies.^13,14^ These observations strengthen the current validation approach that was based on LV remodelling indices. However, the correlation of the DT with other response measures, such as hard clinical endpoints, remains to be investigated in future studies.

Future studies will also investigate the predictive value of the DT at individual patient level, i.e. by virtual optimization of pacing settings (e.g. sites and delays). Investigating the effects of LV lead location was beyond the scope of the current manuscript and may require a prospective study setup where multiple LV lead locations are tested in the same patient. A previous modelling study suggested that LV lead position sensitively determines the distribution of MW, particularly in hearts with left bundle branch block (LBBB) and myocardial scar, where a balanced position remote from both the scar and the RV lead was associated with the most homogeneous distribution of MW.^24^

As far as we know, this is the first patient-specific modelling study that calculates the pacing-induced change of septal-to-lateral work imbalance as a predictor of CRT response. Calculation of acute haemodynamic response has been more common and shows conflicting results between modelling studies. In contrast to our findings, acute improvement of LV dP/dt_max_ was predictive for long-term LV reverse remodelling in other personalized heart models.^25,26^ However, another study showed that the simulated shortening of QRS duration, which may be closely related to acute LV dP/dt_max_ improvement,^27^ correlated poorly with LV reverse remodelling in ischaemic LBBB patients.^28^

While our current DT approach relied on mechanical input information only, this approach should be considered in combination with available electrical measurements of the patient. Baseline QRS area has been shown to have a strong association with echocardiographic response to CRT^16,29^ and may e.g. be combined in a multivariate analysis with the degree of septal-to-lateral work reduction in the DT. Another index that quantifies electrical dyssynchrony at baseline is the standard deviation of electrical activation times, which has been shown to be predictive for post-CRT LVEF improvement.^30^ Although regional RV and LV activation times were automatically estimated by the DT algorithm, it should be noted that this activation represents mechanical rather than electrical activation.

Besides the use of electrical measurements to supplement the DT’s response prediction, these measurements could also be integrated into the DT itself. While the current model implementation is not capable to do so, integration of electrical information into the DT to better characterize the patient’s electrical activation pattern could further improve prediction of the effects of pacing. This electrical characterization may especially be important in ischaemic patients that have abnormal conduction in scarred areas.^31^ When also combining such a DT with models of electrical wave propagation to simulate different pacing conditions,^32^ the interaction between the patient’s electrical and non-electrical tissue characteristics may be more closely investigated.

### Clinical implications

The DTs generated by our algorithm reveal the unique myocardial disease substrates of the patient and may thereby support CRT patient selection as well as therapy delivery. In the first place, the DT’s baseline septal-to-lateral work imbalance provides a marker of CRT response potential. Furthermore, identification of hypocontractile segments in the DT that may indicate ischaemia or scar could guide LV lead location during implantation and potentially present an alternative to the use of late gadolinium enhancement magnetic resonance imaging (MRI) techniques.^21,33^ This study focused on the effects of MW since these have been associated with response to CRT. However, more mechanistic insight might be concealed in the estimated DT tissue characteristics. Future research should try to reveal these insights, which may further improve the predictive power of the DT technology for CRT response.

Based on the interaction of the DT with virtual pacing, moreover, more insight into the mechanisms by which a patient does or does not positively respond to CRT can be obtained. This interaction between the DT and pacing intervention may at the same time provide a platform for therapy optimization in individual HF patients. For example, manipulation of virtually paced AV and VV delay settings and lead location in the DT could allow personalization of pacing delivery. In contrast to using default pacemaker settings, or guiding lead location by e.g. haemodynamic optimization during the implantation procedure,^20^ virtual pacing may identify one or several adequate lead locations prior to pacemaker device implantation. Similarly, the technology could facilitate optimization of pacemaker settings during later stages of CRT delivery, potentially based on generation of a new DT at clinical follow-up.

Application of this DT technology requires data obtained using routine echocardiography and could therefore be relatively easily implemented in clinical practice. Prior to being eligible as a clinical tool to support patient selection and therapy delivery, however, further validation of this technology is essential. This validation could explore how potential errors of currently used clinical data propagate in the DT technology and whether other imaging modalities, such as Doppler echo or cine MRI, are of additional or alternative diagnostic value. Generation of the patient’s DT currently consumed between 12 and 24 h and was thereby by far more time consuming than the virtual pacing, which was performed within seconds. Evaluation of different pacing conditions using the current approach would therefore be highly feasible.

### Limitations

The current study did not compare predictivity of pacing-induced septal-to-lateral work imbalance reduction in the DT with already existing indices to predict the effect of CRT. Our primary focus currently was to present a novel methodology and to demonstrate its potential for clinical application. However, future research should investigate whether this DT technology improves clinical decision-making in dyssynchronous HF patients.

Furthermore, our patient population included eight patients with a Class IIa or IIb indication for CRT. These patients demonstrated a relatively low response to CRT in terms of LV reverse remodelling. Although we found a similarly negative result for these patients by virtual pacing of their DT, i.e. a relatively low workload imbalance reduction, it remains essential to test this technology in more patients with a Class IIa or IIb indication for CRT.

Due to the incomplete documentation of personalized AV and VV delay settings as applied in these patients during clinical CRT, we chose to simulate representative population-based AV and VV delay settings as derived from the original studies. This choice may have influenced the correlations obtained in this study. However, we hypothesize that simulation of these general AV and VV delay settings will rather have worsened than improved these correlations.

## Conclusions

We successfully used virtual biventricular pacing interventions in DTs of dyssynchronous HF patients to predict response to CRT. Our results suggest that the reduction of septal-to-lateral work imbalance by virtual pacing in the DT can predict the patient’s post-CRT LV reverse remodelling. This DT approach could prove to be an additional tool in selecting HF patients for CRT and has the potential to provide valuable insights in optimization of CRT delivery.

## Supplementary Material

euae009_Supplementary_Data

## Data Availability

The data underlying this article will be shared on reasonable request to the corresponding author.
