# Supplementary material for: Virtual pacing of a patient’s digital twin to predict left ventricular reverse remodelling after cardiac resynchronization therapy
Source: Europace. 2024 Jan 30;26(1):euae009. doi: 10.1093/europace/euae009 (PMC10825733; doi:10.1093/europace/euae009)
Supplement: euae009_Supplementary_Data [file euae009_supplementary_data.docx]

**SUPPLEMENTARY MATERIAL**

**Virtual Pacing of a Patient’s Digital Twin to Predict Left Ventricular Reverse Remodelling after Cardiac Resynchronization Therapy**

Tijmen Koopsen^1^, Willem Gerrits^2^, Nick van Osta^1^, Tim van Loon^1^, Philippe Wouters^2^, Frits W. Prinzen^3^, Kevin Vernooy^4,5,6^, Tammo Delhaas^1^, Arco J. Teske^2^, Mathias Meine^2^, Maarten J. Cramer^2^, Joost Lumens^1*^

^1^Department of Biomedical Engineering, CARIM Cardiovascular Research Institute Maastricht, Maastricht University, Maastricht, The Netherlands

^2^Department of Cardiology, Division of Heart and Lungs, University Medical Center Utrecht (UMCU), Utrecht, The Netherlands

^3^Department of Physiology, CARIM Cardiovascular Research Institute Maastricht, Maastricht University, Maastricht, The Netherlands

^4^Department of Cardiology, CARIM Cardiovascular Research Institute Maastricht, Maastricht University, Maastricht, The Netherlands

^5^Department of Cardiology, Maastricht University Medical Center (MUMC), Maastricht, The Netherlands

^6^Department of Cardiology, Radboud University Medical Center, Nijmegen, The Netherlands

*Corresponding author:

Dept. of Biomedical Engineering

CARIM School for Cardiovascular Diseases, Maastricht University Medical Center

PO Box 616, 6200 MD Maastricht, The Netherlands

Tel: +31 43 3881666 / +31 641811758 (mobile)

E-mail: [joost.lumens@maastrichtuniversity.nl](mailto:joost.lumens@maastrichtuniversity.nl)

**Digital Twin generation algorithm**

A total of 75 model parameters were personalized to generate the Digital Twin. These 75 parameters included three global parameters and four parameters estimated in eighteen LV wall segments. The four segmental parameters included mechanical activation delay $dT$, reference mid-wall area $A_{w,ref}$, zero-passive stress length $L_{s0,pas}$, and stiffness coefficient $k$. Furthermore, cardiac output $q_{0}$ was personalized, as well as global LV activation duration offset $ADO$ and atrioventricular (AV) delay relative to the model intrinsic delay $\tau_{AV}$. In the section ‘The CircAdapt model of the human heart and circulation’ of this Supplementary material, these parameters are explained further based on the model governing equations.

Parameters were optimized by minimizing the mean squared error $\chi^{2}$, which was defined as the mean squared error in end diastolic volume $\chi_{V_{ED}}^{2}$, ejection fraction $\chi_{EF}^{2}$, 18 strain traces $\chi_{\varepsilon}^{2}$, and 18 strain rate traces $\chi_{\dot{\varepsilon}}^{2}$:

| $\chi^{2}=\frac{\chi_{V_{ED}}^{2}+\chi_{EF}^{2}+\chi_{\varepsilon}^{2}+\chi_{\dot{\varepsilon}}^{2}}{38}$ | (1) |
| --- | --- |

Error contributor $\chi_{V_{ED}}^{2}$ describes the error between modelled and measured end diastolic volume ($V_{ED,mod}$ and $V_{ED,mea}$, respectively) weighted by $\sigma_{V_{ED}}$:

| $\chi_{V_{ED}}^{2}= \left( \frac{V_{ED,mod}-V_{ED,mea}}{\sigma_{V_{ED}}} \right)^{2} ,$ | (2) |
| --- | --- |

Error contributor $\chi_{EF}^{2}$ describes the error between modelled and measured ejection fraction (${EF}_{mod}$ and ${EF}_{mea}$, respectively) weighted by $\sigma_{EF}$:

| $\chi_{EF}^{2}= \left( \frac{{EF}_{mod}-{EF}_{mea}}{\sigma_{EF}} \right)^{2} ,$ | (3) |
| --- | --- |

Normalization constants $\sigma_{V_{ED}}$ and $\sigma_{EF}$ were assumed to be proportional to the measured value and equaled $0.13\cdot V_{ED,mea}$ and $0.14\cdot{EF}_{mea}$, respectively [1].

Error contributor $\chi_{\varepsilon}^{2}$ describes the error between modelled and measured strain ($\varepsilon_{i,mod}$ and $\varepsilon_{i,mea}$, respectively) weighted by $\sigma_{\varepsilon_{i}}$ and averaged over all data points $n_{dp}$.

| $\chi_{\varepsilon}^{2}=\sum_{i=1}^{n_{seg}} \left( \frac{1}{n_{dp}}\sum_{k=1}^{n_{dp}} \left( \frac{\varepsilon_{i,mod}(k)-\varepsilon_{i,mea}(k)}{\sigma_{\varepsilon_{i}}} \right)^{2} \right) ,$ | (4) |
| --- | --- |

Error contributor $\chi_{\dot{\varepsilon}}^{2}$ describes the error between modelled and measured strain rate ($\varepsilon_{i,mod}$ and $\varepsilon_{i,mea}$, respectively) weigthed by $\sigma_{\varepsilon_{i}}$ and averaged over all data points $n_{dp}$.

| $\chi_{\dot{\varepsilon}}^{2}=\sum_{i=1}^{n_{seg}} \left( \frac{1}{n_{dp}}\sum_{k=1}^{n_{dp}} \left( \frac{\dot{\varepsilon}_{i,mod}(k)-\dot{\varepsilon}_{i,mea}(k)}{\sigma_{\dot{\varepsilon_{i}}}} \right)^{2} \right) .$ | (5) |
| --- | --- |

|  |  |  |
| --- | --- | --- |
|  |  |  |
|  | |  |
|  | |  |

Only strain from mitral valve closure till 10% global re-lengthening + 50 ms was included in the cost function, thereby excluding late diastolic strain. Simulated strain signals $\varepsilon_{i,mod}$ were obtained by scaling simulated fiber strain $\varepsilon_{i,mod,f}$ to the amplitude of the longitudinal strain measurements of the patient:

| $\varepsilon_{i,mod,f}\left( t \right)=\frac{L_{s,i}\left( t \right)-L_{s,i}\left( t_{0} \right)}{L_{s,i}\left( t_{0} \right)}\cdot100\%$, | (6) |
| --- | --- |
|  |  |
| $\varepsilon_{i,mod}=\frac{\varepsilon_{glob,mea}}{\varepsilon_{glob,mod,f}}\cdot\varepsilon_{i,mod,f}$. | (7) |

Here, $L_{s,i}(t)$ is the sarcomere length of segment $i$ at time $t$, while $t_{0}$ is the timing of mitral valve closure. Furthermore, $\varepsilon_{glob,mea}$ and $\varepsilon_{glob,mod,f}$ are the measured and simulated peak values of the global strain signal, i.e., the average strain signal of all 18 LV segments. Simulated strains and strain rates were also resampled to the sampling frequency of $\varepsilon_{mea}$. Measurement uncertainties $\sigma_{\varepsilon}$ and $\sigma_{\dot{\varepsilon}}$ were chosen to equal 2% and 20%/s, respectively. Cycle time within the model ($tCycle$) was fixed to the average cycle time $t_{cycle}$ of all three echocardiographic acquisitions.

The optimization algorithm used was dynamic multi-swarm particle swarm optimization (DMS-PSO) [2]. A total of 60 particles were used, subdivided into 20 swarms of three particles. Every 20 iterations, swarms were randomly regrouped. The initial particle positions were determined by performing 1,000 Monte Carlo (MC) simulations using the ranges shown in Supplementary Table 1. To prevent non-realistic simulations, however, additional restrictions on the severity of electrical dyssynchrony, global LV contractility and stiffness were imposed. The 60 particles with the lowest cost function values $\chi^{2}$ were selected as initial particle positions.

During iterations of DMS-PSO, extended parameter boundaries were used (Supplementary Table 1) as compared to those for MC simulations to improve algorithm performance. For particles outside these boundaries, the cost function was infinite. Particle velocities were limited to 25% of the input space width to prevent particles from oscillating outside the input space. DMS-PSO was stopped when normalized particle energies were lower than 10^-4^, meaning no parameter changed more than 1% of its input space width within one iteration, or when a maximum number of 2,000 iterations were completed.

**The CircAdapt model of the human heart and circulation**

The CircAdapt model of the human heart and circulation is a closed-loop lumped-parameter model which simulates beat-to-beat hemodynamics and mechanics of the heart and blood vessels [3]. In this paper, we used the model build as described by Walmsley et al.[4] which later was implemented in c++ to improve performance [5]. The pulmonary and systemic circulation are modeled using a three-element model of resistive wave impedance, compliance, and peripheral resistance [6]. Cardiac walls are modelled as spherical shells, and the left and right ventricular walls are coupled through the interventricular septum in the TriSeg geometry [7]. In this geometry, wall tension $T_{w}$ was linearized based on the mid-wall surface area $A_{w}$:

| $T_{w}\left( t \right)=\frac{V_{w}\cdot\sigma_{f}(t)}{2\cdot A_{w}(t)}$ | (8) |
| --- | --- |

Here, $V_{w}$ is the wall volume, while $\sigma_{f}(t)$ represents myofiber stress which is a function of natural myofiber strain $\varepsilon_{f}(t)$. Furthermore, $z$ is a dimensionless curvature parameter which is closely related to the ratio of wall thickness to radius of curvature. The relation between $\varepsilon_{f}(t)$ and $A_{w}(t)$ is determined by a reference wall area $A_{w,ref}$, which is defined as the area at a reference sarcomere length $L_{s,ref}=2$ $\mu m$:

| $\varepsilon_{f}(t)=\frac{1}{2}\ln\left( \frac{A_{w}(t)}{A_{w,ref}} \right)$ | (9) |
| --- | --- |

Natural myofiber strain $\varepsilon_{f}(t)$ determines sarcomere length $L_{s}(t)$ by:

| $L_{s}(t)=L_{s,ref}\cdot e^{\varepsilon_{f}(t)}$ | (10) |
| --- | --- |

Active and passive myofiber stress $\sigma_{f,act}(t)$ and $\sigma_{f,pas}(t)$ are calculated based on $L_{s}(t)$ using a three-element Hill contraction model. In this model, $\sigma_{f,act}(t)$ is calculated by:

| $\sigma_{f,act}(t)=S_{f,act}\cdot C(t)\cdot\left( L_{si}\left( t \right)-L_{si,0} \right)\cdot\frac{L_{se}(t)}{L_{se,iso}}$ | (11) |
| --- | --- |

where $S_{f,act}$ scales active stress development, while $C(t)$ is a state variable representing the density of cross-bridge formation. Furthermore, $L_{si}\left( t \right)$ represents the contractile element length, which has a zero-active stress reference length $L_{si,0}=1.51 \mu m$. Variable $L_{se}\left( t \right)$ is the length of the series elastic element, which is scaled with a reference length of $L_{se,iso}=0.04 \mu m$ defined at the onset of isovolumetric contraction. Variable $C(t)$ is described by the following differential equation:

| $\frac{dC}{dt}=\frac{1}{\tau_{r}}C_{L}\left( L_{si}(t) \right)\cdot F_{rise}\left( t \right)-\frac{1}{\tau_{d}}\cdot C(t)\cdot g(X)$ | (12) |
| --- | --- |

In this differential equation, the time constants $\tau_{r}$ and $\tau_{d}$ scale the rise and decay of $C(t)$, respectively. The function $C_{L}\left( L_{si} \right)$ describes the increase of force development with sarcomere length, and $F_{rise}\left( t \right)$ describes the time-dependent rise of mechanical activation. The term $g(X)$ causes a delay in the decay of contractility. It holds:

| $X=\frac{t_{c}-t_{A}}{\tau_{d}}$ | (13) |
| --- | --- |

with $t_{c}=t-t_{act}$ the time elapsed since onset of activation $t_{act}$, and $t_{A}$ the duration of activation. This duration of activation is determined by a length-independent offset $ADO$ as well as a length-dependent parameter $LDAD$:

| $t_{A}=\left( ADO+LDAD\cdot\frac{L_{si}(t)}{L_{si,0}} \right)\cdot T_{a}$ | (14) |
| --- | --- |

where $T_{a}$ is a factor scaling the contraction duration.

Passive stress $\sigma_{f,pas}(t)$ includes two components:

| $\sigma_{f,pas}(t)=\sigma_{f,tit}(t)+\sigma_{f,ECM}(t)$ | (15) |
| --- | --- |

where $\sigma_{f,tit}(t)$ is the stress that arises from the myocytes themselves due to intracellular structures such as titin, and $\sigma_{f,ECM}(t)$ is the stress resulting from the extracellular matrix (ECM). The intracellular component $\sigma_{f,tit}(t)$ is defined by:

| $\sigma_{f,tit}\left( t \right)=0.01\cdot S_{f,act}\cdot\left( \lambda_{p}{(t)}^{20/3}-1 \right)$ | (16) |
| --- | --- |

while the ECM component $\sigma_{f,ECM}(t)$ is calculated as:

| $\sigma_{f,ECM}(t)=S_{f,pas}\cdot\left( \lambda_{p}{(t)}^{k}-1 \right)$ | (17) |
| --- | --- |

Here, $S_{f,pas}$ is a scaling factor, while $k$ determines the non-linearity of the relation between $\lambda_{p}{(t)}^{k}$ and $\sigma_{f,ECM}(t)$. Variable $\lambda_{p}(t)$ is calculated as:

| $\lambda_{p}(t)=\frac{L_{s}(t)}{L_{s0,pas}}$ | (18) |
| --- | --- |

with $L_{s}(t)$ being sarcomere length and $L_{s0,pas}$ the zero-passive stress length.

Supplementary Table 1: Overview of the 75 parameters personalized and their boundaries used during Monte Carlo (MC) simulations and dynamic multi-swarm particle swarm optimization (DMS-PSO). glob, global LV parameter; seg, segmental LV parameter; S, septum; LVfw, LV free wall; $t_{cycle,mea}$, measured cycle time.

| **Parameter** | **Number of parameters** | **Physiological meaning** | **Unit** | **Lower bound (MC)** | **Upper bound (MC)** | **Lower bound (DMS-PSO)** | **Upper bound (DMS-PSO)** |
| --- | --- | --- | --- | --- | --- | --- | --- |
| $q0$ | 1 | Cardiac output | L/min | 2.1 | 5.5 | 1 | 20 |
| $\tau_{AV}$ | 1 | Atrioventricular delay relative to model intrinsic delay | s | -0.050 | 0.130 | -0.100 | 0.200 |
| $ADO$ (glob) | 1 | Activation duration offset | s | 0.52 | 0.78 | 0 | 2*$t_{cycle,mea}$ |
| $dT$ (seg) | 18 | Mechanical activation delay | s | -0.030 (S)  0.000 (LVfw) | 0.060 (S),  0.120 (LVfw) | -0.060 (S)  -0.060 (LVfw) | 0.120 (S),  0.200 (LVfw) |
| $A_{w,ref}$ (seg) | 18 | Reference wall area | cm^2^ | 16/3 | 35/3 | 1.6 (S)  1.6 (LVfw) | 24.4 (S)  24.5 (LVfw) |
| $L_{s0,pas}$ (seg) | 18 | Zero-passive stress length | µm | 1.63 | 1.99 | 0 | 3 |
| $k$ (seg) | 18 | Stiffness coefficient | - | 6.4 | 36 | 0 | 100 |
|  | 75 |  |  |  |  |  |  |

[1] J. E. Otterstad, G. Froeland, M. St John Sutton, and I. Holme, “Accuracy and reproducibility of biplane two-dimensional echocardiographic measurements of left ventricular dimensions and function,” *European Heart Journal*, vol. 18, no. 3, pp. 507–513, 1997, doi: 10.1093/oxfordjournals.eurheartj.a015273.

[2] J. J. Liang and P. N. Suganthan, “Dynamic multi-swarm particle swarm optimizer,” *Proceedings 2005 IEEE Swarm Intelligence Symposium*, pp. 124–129, 2005.

[3] T. Arts, T. Delhaas, P. Bovendeerd, X. Verbeek, and F. W. Prinzen, “Adaptation to mechanical load determines shape and properties of heart and circulation: the CircAdapt model,” *American Journal of Physiology-Heart and Circulatory Physiology*, vol. 288, no. 4, pp. H1943–H1954, 2004, doi: 10.1152/ajpheart.00444.2004.

[4] J. Walmsley *et al.*, “Fast Simulation of Mechanical Heterogeneity in the Electrically Asynchronous Heart Using the MultiPatch Module,” *PLoS Comput Biol*, vol. 11, no. 7, p. e1004284, Jul. 2015, doi: 10.1371/journal.pcbi.1004284.

[5] N. van Osta *et al.*, “Parameter subset reduction for patient-specific modelling of arrhythmogenic cardiomyopathy-related mutation carriers in the CircAdapt model,” *Philosophical Transactions of the Royal Society A: Mathematical, Physical and Engineering Sciences*, vol. 378, no. 2173, p. 20190347, Jun. 2020, doi: 10.1098/rsta.2019.0347.

[6] T. Arts, K. Reesink, W. Kroon, and T. Delhaas, “Simulation of adaptation of blood vessel geometry to flow and pressure: Implications for arterio-venous impedance,” *Mechanics Research Communications*, vol. 42, pp. 15–21, 2012, doi: 10.1016/j.mechrescom.2011.10.005.

[7] J. Lumens, T. Delhaas, B. Kirn, and T. Arts, “Three-wall segment (TriSeg) model describing mechanics and hemodynamics of ventricular interaction,” *Annals of Biomedical Engineering*, vol. 37, no. 11, pp. 2234–2255, 2009, doi: 10.1007/s10439-009-9774-2.
